# Annotations

**Published:** 1900-03-10

**Authors:** 


### Annotations.

### Look Before You Leap.

The extraordinary desire manifested in all ranks of society to get to South Africa at any price and in almost any capacity is responsible for some curious results. Perhaps the authorities may not be exclusively to blame for anomalies which at first sight seem to indicate gross carelessness in administration; but we would strongly urge people who intend to volunteer to look for themselves before they leap, and inquire carefully as to the duties which will fall to their lot before they jump at the bait of getting "somehow or other" to the front. An indignant father writes to the papers to complain of the treatment that befell his son, a medical student, who had gone through his curriculum and had been signed up for his final. This youth, it appears, being a member of the V.M.S.C., volunteered for South Africa, and is now stationed at Woolwich waiting for embarkation, together with other medical volunteers, and a large number of the St. John Ambulance Corps. Here comes the odd part of the business. These St. John men, who rank as first-class orderlies, are excused from the menial work which the medical students, who rank as third-class orderlies, have to perform, and thus we have the ridiculous spectacle of medical students, who have been through the hospitals and are the very best men possible for undertaking the highest posts that could be found for them as dressers in the hospitals, being made to scrub the floors, black-lead the grates, clean the non-commissioned officers' quarters, &c., while the St. John men, just ordinary people who have been taught a little first aid, are standing by as first-class orderlies. The whole thing would be too laughable were it not that it shows how dreadfully little common sense seems to be available in arranging these affairs. Of course, it is to be presumed that these medical students might have known what their position would be if, on joining the V.M.S.C., they had chosen to inquire. But that does not take away the absurdity of the fact that while the War Office is asking for medical students to go out to the hospitals, it is using such as it possesses in doing the ordinary menial work, from which the ambulance men as "first-class orderlies" are excused.

### The Doctor's Bill.

Under the above heading our sometime rather cynical contemporary, the *Practitioner*, has a paragraph dealing with the cost to the British taxpayer involved in the despatch of seven consulting surgeons to South Africa, each receiving pay at the rate of £5,000 a year. The appointment of these gentlemen was, it is said, due in the first instance to the clamour of certain "leaders of society," who were naturally anxious that the high-born warriors in whom they were specially interested should have the best skill procurable if they were wounded. In regard to the consultants who were appointed, it is remarked that, although all of them were surgeons of approved skill, of some it might be said, without the least reflection on their professional skill, that they had blushed unseen within the walls of their hospitals before this greatness was thrust upon them The following little story is told of War Office routine, which suggests how freely money is apt to slip away in time of war. One of the gentlemen lately sent out is said to have asked that he might be allowed to take out a junior surgeon as assistant. This very natural request, for some mysterious reason, greatly exercised the mind of the War Office, and finally the application was refused on the ground that it was impossible officially to recognise such an anomaly as an "assistant." So the fortunate junior was appointed a consulting surgeon, on the same footing as the others, with a salary of £5,000 a year! Truly a pretty little tale.

### Electric Power Distribution.

It may seem outside our duty to intervene in the various economic questions which arise from time to time in regard to the conduct of the great industries of this country, but now and again problems come to the front which, although on the surface apparently purely industrial, have such intimate bearings upon sanitation as to render them subjects of the greatest importance to all who concern themselves with the public health. Among such is that of the distribution of energy in the form of electricity. It is a curious thing that the proposals which are now being made for the establishment of enormous electric works in the neighbourhood of the coal pits, where electricity can be produced at an extremely cheap rate, and for its distribution to those parts where light and power are required, should meet with opposition in those quarters which are supposed to represent the feelings of the working men, seeing how enormously the wide distribution of power must conduce to commercial prosperity, and especially seeing how largely it must directly tend to improve the position of the workers. In a country like England, which depends for its food upon its manufactures, it is the fate of the masses to live where power is to be found. Hence industrial life is crowded by the side of rivers, in the neighbourhood of coal mines, and in great railway centres. Coal is the great food on which English industrialism lives, and under present circumstances the working classes are condemned to live amid the dirt that coal produces, and cut off from the sunlight by the cloud which its use entails. How different industrial life might be if power were produced far away, and were brought into the manufacturing centres, partly in the form of electricity produced at the pit-mouth, partly in the form of gas made in places devoted to disagreeable processes! Electric traction without the nuisance of electric works, gas fires without the nuisance of coal smoke, mills without chimneys, power available everywhere by merely turning on a switch, would not only give us back again the clear light of heaven in our towns, but might make it possible once again to establish country industries, so that men should have access to that great element of happiness—variety of work. If electric power were once available mills—smokeless mills—would arise in country places all along the tracks of the electrical conduits, and every mill so arising would be an influence cutting at the root of that overcrowding which is the leading cause of the physical deterioration of the working class population. If electric power distribution means smokeless towns and country mills by all means let us have it.

